# Worldwide scientific productions with immunotherapy of sepsis: a bibliometric analysis

**DOI:** 10.7717/peerj.7116

**Published:** 2019-06-17

**Authors:** Ronghao Wan, Lei Li, Chenwei Xing, Ronggang Peng, Liang Gao

**Affiliations:** 1 Department of Neurosurgery, Tenth People’s Hospital Affiliated to Tongji University, Shanghai, China; 2 Department of Medical Administration, Tenth People’s Hospital Affiliated to Tongji University, Shanghai, China; 3 Department of Cardiology, Tenth People’s Hospital Affiliated to Tongji University, Shanghai, China

**Keywords:** Sepsis, Immunotherapy, Bibliometric analysis

## Abstract

**Background:**

Sepsis represents a significant healthcare problem worldwide and causes a high number of deaths every year but remains to be fully understood. During and after sepsis, the host immune response is complex and involves an initial excessive host inflammatory response to infection that is closely related to tissue damage and leads to organ failure. Over the past three decades, immunotherapy for sepsis has vastly improved, but in this area, the most influential articles, journals, authors, and countries have not yet been completely summarized and analyzed.

**Objective:**

Performed a bibliometric analysis on all the articles concerning immunotherapy for sepsis from 1962 to 2019 was our objective, and we also explored the potential correlations between publications of different countries and their gross domestic product (GDP).

**Methods:**

All articles about immunotherapy for sepsis were extracted from the Scopus database and analyzed. We also retrieved GDP data from all the countries that have published information from the World Bank.

**Results:**

In summary, we have retrieved 1,483 related articles from the Scopus database starting from the first publication on immunotherapy for sepsis in 1962 through March 16, 2019. Over the past decade, the number of the articles published has increased year by year to reach 866 in total, which accounts for about 58% of all publications, with 2017 being the most prolific year when 179 articles were published. The US published 604 articles (41%), followed by China (*n* = 163, 11%), and Germany (*n* = 158, 11%). In terms of publishing media, the journal that published the highest number of the articles was Journal of Critical Care Medicine with 65 articles (4%), followed by Shock with 55 articles (4%), and Critical Care with 35 articles (2%). There was a strong correlation between the GDP of the different countries and their publication numbers (*r* = 0.811, *P* < 0.001).

**Conclusions:**

Our present study analyzed all types of articles concerning immunotherapy for sepsis over the past 57 years and countries with high GDP tends to make more contributions to the medical field of this field. In the meantime, these studies highlight the importance of immunotherapy in the treatment of sepsis patients. The recognition of the historical status and development trend of this field can promote inter-agency cooperation, guide future research, and ultimately provide the basis for clinical practice guidelines.

## Introduction

Sepsis is still a major healthcare problem to be fully recognized worldwide and causes a high number of deaths each year. The current estimates are about 30 million cases and six million deaths every year (Fleischmann et al., 2016; Reinhart et al., 2017). The main reason for this is that there is still no effective specific anti-sepsis treatments. Thus, management of patients with sepsis depends mainly on early recognition allowing correct therapeutic measures to be started rapidly including administration of appropriate antibiotics, source control measures when necessary, and resuscitation with intravenous fluids and vasoactive drugs when needed (Cohen et al., 2015). Thankfully, researchers have fully recognized that due to the dynamic nature of the disease, such as the cytokine profile changes with time, the underlying infection can move from being localized to disseminated, and the immunological profile can change from being proinflammatory to more immunosuppressed (Deutschman & Tracey, 2014; Seok et al., 2013). Then, in the third consensus on sepsis or sepsis-3, it is defined as a life-threatening condition of organ dysfunction caused by a dysregulated host’s immune response to infection (Singer et al., 2016). The question remaining is whether therapeutic interventions that target specific immune process mechanisms implicated in the pathophysiological changes of sepsis might further improve therapeutic effects and save additional lives. The World Health Organization announced sepsis as a global health priority on May 26, 2017 by taking the resolution to improve the prevention, diagnosis, and management of this deadly disease (Reinhart et al., 2017). Similarly, the number of articles related to immunotherapy of sepsis reached a peak in 2017. Generally speaking, the number of published articles and citations are essential indicators of the scientific productivity of a country. Bibliometric analysis is usually used to describe the importance of published articles and show research trends on specific topics which can guide future research and provide evidence for clinical practice (Alfaifi et al., 2018).

Bibliometric analysis has been widely used in medical research. Besides critical care medicine (Zhang et al., 2018), it also includes traumatology (Dokur & Uysal, 2018), emergency medicine (Prats et al., 2018), neurosurgery (Alfaifi et al., 2018), oncology (Cabral, da Graca Derengowski Fonseca & Mota, 2018), gynecology (Huang et al., 2018), pediatrics (McDowell et al., 2017), and psychology (Tur-Porcar et al., 2018) and so on.

To date, bibliometric analysis has not been applied to the field of immunotherapy for sepsis. Therefore, we focus on exploring the characteristics of scientific research productivity in this field. In this way, we can understand the research trends and define some leading countries, which will help us strengthen international cooperation in this field, promote the common progress of the discipline, and ultimately benefit patients.

A comprehensive bibliometric analysis absolutely can’t be separated from a reliable and complete database. The Scopus database is the world’s largest abstract and citation database of peer-reviewed literature, with more than 19,500 peer-reviewed journals across various disciplines being indexed, covering research topics across all scientific and technical disciplines, ranging from medicine and social sciences to arts and humanities (Gasparyan, Ayvazyan & Kitas, 2013). For example, all MEDLINE-indexed journals are covered by Scopus, and it is also one of the commonly used databases in the analysis of medical research results (Gasparyan, Ayvazyan & Kitas, 2013; Kokol & Vosner, 2018). Scopus features smart tools to track, analyze and visualize research. What’s more, daily data updates enable us to obtain current, reliable data. These salient features are entirely in line with our needs, so we chose it for our research.

## Materials and Methods

### Literatures searching

On March 16, 2019, we performed advanced retrieval in the Scopus database with no restriction on the language or time. We’ve summed up eight main search terms, including “sepsis,” “severe sepsis,” “pyemia,” “pyohemia,” “pyaemia,” “septicemia,” “blood poisoning,” and “immunotherapy.” The fuzzy matching mode was selected for each search term to ensure the completeness and comprehensiveness of the retrieval results. Finally, our retrieval type was “TITLE (sepsis) OR TITLE (severe sepsis) OR TITLE (pyemia) OR TITLE (pyohemia) OR TITLE (pyaemia) OR TITLE (septicemia) OR TITLE (blood poisoning) AND ALL (immunotherapy).” With this approach, we recorded and reviewed 1,483 articles. Our title-specific search for sepsis was intended to make the results more representative and to avoid the proliferation of search results since sepsis is a life-threatening condition of organ dysfunction, concerning a variety of diseases.

### Bibliometrics analysis

We used the analysis tool in the Scopus database to record the basic information of articles, such as distributions in different medical departments, publication titles, authors, countries, and journals. We took the number of available literature to be an indicator of the number of scientific research achievements. The number of citations is considered an important index of the attention and influence degree of the published papers (Fardi et al., 2011). The primary outcomes were the number of articles attributed to each year and each country, as well as the citations attributed to each paper. In terms of the number of literature published, we focused on the top 10 authors, journals and countries, as well as the top 10 most-cited articles. These were summarized by the descriptive analysis. Secondly, we explored and evaluated the potential correlations between the number of publications of different countries and their gross domestic product (GDP). Based on the World Bank national accounts data and the Organization for Economic Co-operation and Development National Accounts data files, we obtained the data of these different countries’ GDP in 2017.

### Statistical analysis

All statistical analyses were performed using SPSS software version 20.0 (SPSS Inc, Chicago, IL, USA). The Spearman rank correlation test determined the statistical significance of the correlation. |*r*| < 0.3 indicates a weak correlation, 0.3< |*r*| < 0.5 indicates low correlation, 0.5 < |*r*|< 0.8 indicates significant correlation, 0.8 < |*r*| < 1 indicates high correlation, *r* > 0 indicates two variables are positively correlated, *r* < 0 indicates two variables negatively correlated, *r* = 0 indicates two variables are irrelevant. *P* < 0.01 was considered to be statistically significant.

## Results

### General information

**Figure 1:**
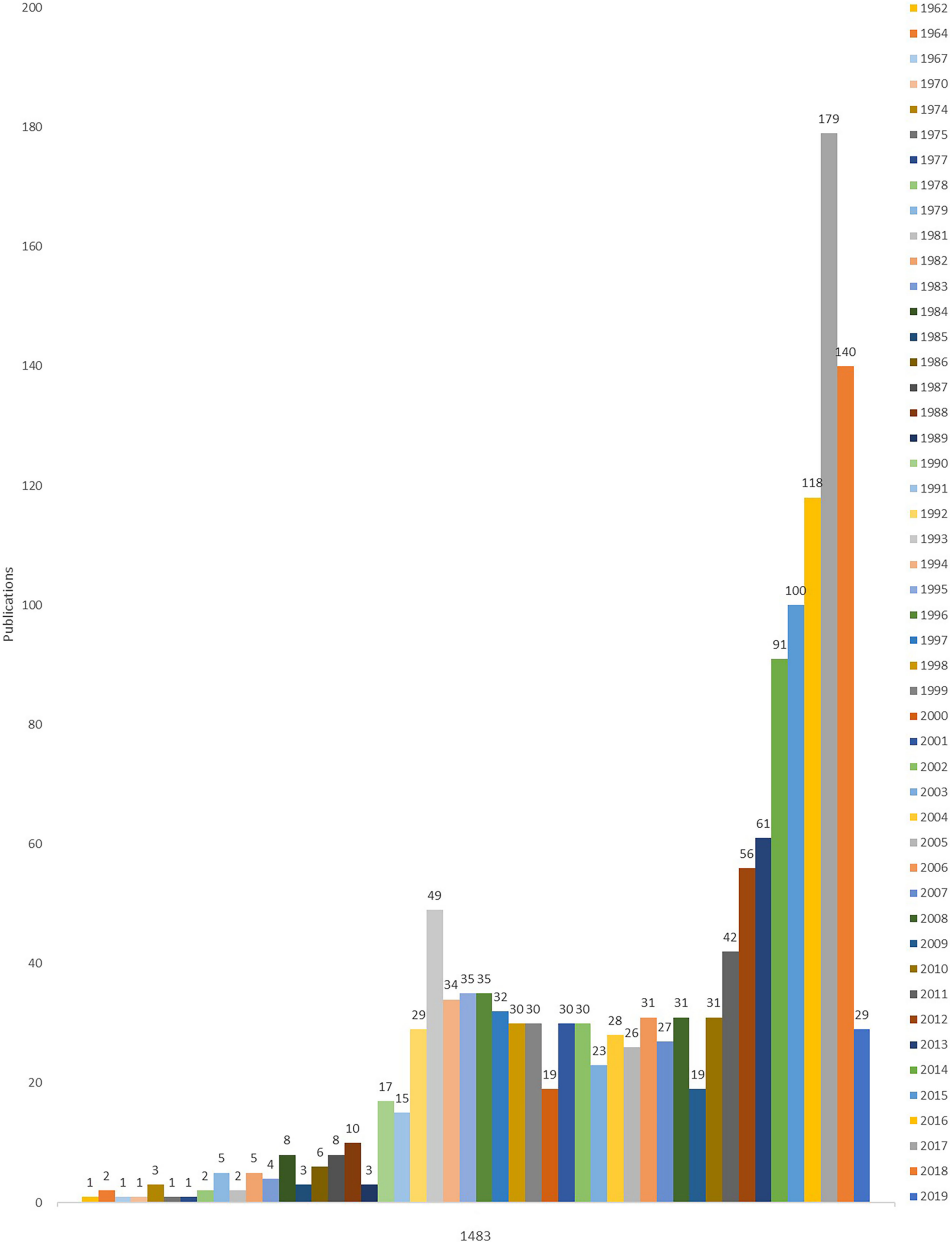
The distribution of publications about immunotherapy for sepsis on the Scopus database in each year. Note: 1963, 1965, 1966, 1968, 1969, 1971, 1972, 1973, 1976, 1980 published the number of articles is zero, so it is not presented in the diagram.

We checked the Scopus database and found 1,483 pieces of relevant documents, which included Article, Review, Editorial, Conference Paper, Book Chapter, Letter, Note, Short Survey, and Article in Press. Among them, the top 10 of all the articles were selected for summary and analysis (Fig. 1). The number of articles published in 2009–2019 (*n* = 866) increased by 156% compared to the number of articles published in 1989–2008 (*n* = 554) and by 315% compared to the number of articles published in 1999–2008 (*n* = 275). As of March 16, 2019, the total citation of all the articles was 48,125, and the number of median citations was seven, which indicated a relatively attention and influence degree of overall literature. The highest cited article was “Surviving sepsis campaign: International guidelines for management of severe sepsis and septic shock: 2012” (times cited = 3,435), followed by “The third international consensus definitions for sepsis and septic shock (sepsis-3)” with 2,859, and “The pathophysiology and treatment of sepsis” with 2,711. The top 10 most-cited articles were shown in Table 1. The number of their median citations is 1,175.

**Table 1:** Top 10 most-cited articles about immunotherapy of sepsis.

Title	Publication year	Journals (citescore 2017)	First author	Country	Scopus citations
Surviving sepsis campaign: International guidelines for management of severe sepsis and septic shock: 2012	2013	Critical Care Medicine (3.24)	Dellinger, Richard Phillip	US	3,435
The third international consensus definitions for sepsis and septic shock (sepsis-3)	2016	JAMA (7.30)	Singer, Mervyn	UK	2,859
The pathophysiology and treatment of sepsis	2003	New England Journal of Medicine (14.75)	Hotchkiss, Richard S.	US	2,711
Surviving sepsis campaign: International guidelines for management of severe sepsis and septic shock, 2012	2013	Intensive Care Medicine (3.99)	Dellinger, Richard Phillip	US	2,409
Recombinant Human Interleukin 1 Receptor Antagonist in the Treatment of Patients With Sepsis Syndrome: Results From a Randomized, Double-blind, Placebo-Controlled Trial	1994	JAMA (7.30)	Fisher, Charles J.	US	842
Immunosuppression in patients who die of sepsis and multiple organ failure	2011	JAMA (7.30)	Boomer, Jonathan S.	US	666
Novel strategies for the treatment of sepsis	2003	Nature Medicine (16.81)	Riedemann, Niels Christoph	Germany	635
Sepsis-induced immunosuppression: From cellular dysfunctions to immunotherapy	2013	Nature Reviews Immunology (17.43)	Hotchkiss, Richard S.	US	593
A Controlled Clinical Trial of E5 Murine Monoclonal IgM Antibody to Endotoxin in the Treatment of Gram-Negative Sepsis	1991	JAMA (7.30)	Greenman, Richard L.	US	583
Confirmatory interleukin-1 receptor antagonist trial in severe sepsis: A phase III, randomized, double-blind, placebo-controlled, multicenter trial	1997	Critical Care Medicine (3.24)	Opal, Steven M.	US	553

### Distributions

The country with most publication counts was the United States of America (USA) which has published 604 articles (41%), and the second productive country was China with 163 (11%), followed by Germany (*n* = 158, 11%) (Table 2).

**Table 2:** The top 10 countries with publications about immunotherapy for sepsis and ranked by the publication numbers.

Countries/Territories	Record count	% of 1,483
US	604	41
China	163	11
Germany	158	11
UK	100	7
France	81	5
Netherlands	69	5
Canada	47	3
Italy	40	3
Australia	39	3
Japan	39	3

Combining Table 2 with Table 3, we can clearly see that the combined scientific output of the first three countries exceeds 50% of the total output. Moreover, these gains are concentrated in countries with higher GDP.

**Table 3:** The data of 62 different countries with publications and ranked by GDP (current US $) in 2017.

Countries	GDP (million $)
US	19,390,604
China	12,237,700
Japan	4,872,137
Germany	3,677,439
UK	3,622,434
India	2,597,491
France	2,582,501
Brazil	2,055,506
Italy	1,934,798
Canada	1,653,043
Russian Federation	1,577,524
Korea, Rep.	1,530,751
Australia	1,323,421
Spain	1,311,320
Mexico	1,149,919
Indonesia	1,015,539
Turkey	851,102
Netherlands	826,200
Saudi Arabia	683,827
Switzerland	678,887
Argentina	637,590
Taiwan	579,302^a^
Sweden	538,040
Poland	524,510
Belgium	492,681
Thailand	455,221
Iran, Islamic Rep.	439,514
Austria	416,596
Norway	398,832
United Arab Emirates	382,575
Israel	350,851
South Africa	349,419
Ireland	333,731
Denmark	324,872
Singapore	323,907
Malaysia	314,500
Philippines	313,595
Colombia	309,191
Pakistan	304,952
Chile	277,076
Finland	251,885
Egypt, Arab Rep.	235,369
Portugal	217,571
Czech Republic	215,726
Romania	211,803
New Zealand	205,853
Greece	200,288
Kazakhstan	159,407
Hungary	139,135
Ethiopia	80,562
Panama	61,838
Bulgaria	56,832
Croatia	54,849
Lithuania	47,168
Serbia	41,432
Latvia	30,264
Nepal	24,472
Cyprus	21,652
Zimbabwe	17,846
Georgia	15,081
Armenia	11,537
Cuba	8,713^b^

**Notes:**

a We obtained the data of GDP of Taiwan from CIA “The World Factbook” rather than from World Bank.

b We obtained the data of GDP of Cuba in 2015 rather than in 2017.

The journal that published the highest number of original articles was Critical Care Medicine with 65 (4%), followed by Shock (*n* = 55, 4%), and Critical Care (*n* = 35, 2%). Their median CiteScore is 4.23. (Table 4).

**Table 4:** Top 10 journals about immunotherapy for sepsis ranked by the number of the publications.

Journals	CiteScore 2017	Record count	% of 1,483
Critical Care Medicine	3.24	65	4
Shock	2.75	55	4
Critical Care	4.53	35	2
PLOS ONE	3.01	30	2
American Journal of Respiratory and Critical Care Medicine	5.10	25	2
Journal of Immunology	4.57	23	2
Intensive Care Medicine	3.99	21	1
Frontiers in Immunology	5.62	20	1
Journal of Infectious Diseases	4.47	19	1
Infection and Immunity	3.43	17	1

The most prolific author was Opal, S.M., who has 33 articles (2%), followed by Hotchkiss, R.S. (*n* = 29, 2%), and Vincent, J.L. with 20 (1%). (Table 5).

**Table 5:** The top 10 authors with publications about immunotherapy for sepsis and ranked by the publication numbers.

Authors	Record count	% of 1,483
Opal, S.M.	33	2
Hotchkiss, R.S.	29	2
Vincent, J.L.	20	1
Monneret, G.	17	1
Coopersmith, C.M.	16	1
Dunn, D.L.	16	1
Moldawer, L.L.	15	1
Shankar-Hari, M.	15	1
Van Der Poll, T.	14	1
Venet, F.	14	1

Moreover, based on the data of the World Bank, we obtained the data from different countries’ GDP in 2017 (Table 3) and ranked by their GDP. Spearman rank correlation analysis revealed that there was a highly positive correlation between GDP of different countries and the publication numbers (*r* = 0.811, *P* < 0.001).

## Discussion

After evaluating 57 years of research, we have clearly recognized that sepsis is associated with the activation of multiple inflammatory pathways and that sepsis can also lead to an immunosuppressive state. That is to say, some patients with “sepsis” clearly show evidence of hyperinflammation (e.g., meningococcal shock), other patients or immunosuppressive condition that could leave patients more susceptible to secondary nosocomial infections (Van der Poll, 2001). Therefore, as an important adjuvant therapy, immunomodulatory therapy has attracted more and more attention from sepsis researchers. Over the past 10 years, medical workers have made considerable achievements in the field and the description of sepsis-induced immune dysfunctions has improved (Venet & Monneret, 2018). Our bibliometric analysis demonstrated that the number of articles published in the last decade has a sharp increase of 315% compared with those published in the previous decade. In addition, the number of median citations of all the articles was five. We have identified a variety of mechanisms that cause immunosuppression in patients with sepsis, including vast apoptosis-induced depletion of lymphocytes and dendritic cells, decreased expression of the cell-surface antigen presenting complex HLA-DR, and increased expression of the negative costimulatory molecules programmed death 1 (PD-1), cytotoxic T-lymphocyte associated antigen 4, and B- and T-lymphocyte attenuator, and their corresponding ligands (e.g., PD-1 ligand [PD-L1]), as well as the numbers of regulatory T cells and myeloid-derived suppressor cells are increased, and there is a shift from a phenotype of inflammatory type 1 helper T (Th1) cells to an antiinflammatory phenotype of type 2 helper T (Th2) cells characterized by the production of interleukin-10 (Hotchkiss & Opal, 2010). What’s more, the management of patients with sepsis will necessitate more rigorous approaches to disease description and stratification (Abraham, 2016; Bone, 1996; Fink & Warren, 2014) and for the immunotherapy to be rational and effective, the immunocompetence of a patient at a given stage of disease has to be evaluated (Prucha, Zazula & Russwurm, 2017). For the future, we believe that the prevention of sepsis-induced immunosuppression, or its treatment if it occurs, is a research priority (Hotchkiss & Opal, 2010). At the same time, investment in both basic and translational clinical science should be increased with the aim of achieving two goals: to identify new treatment targets and strategies, focusing on those that can help patients the world over, including patients with sepsis who live in low-income and middle-income countries; and to develop the application of the principles of personalized medicine to the treatment of sepsis (Cohen et al., 2015). These goals cannot be achieved without the support of the World Health Organization, as well as increased funding from governments and the efforts of researchers (Reinhart et al., 2017). And the findings suggested a booming development of immunotherapy of sepsis with active research activity.

Now we can see the extensive use of bibliometric analysis to detect worldwide scientific productions (Alfaifi et al., 2018; Cabral, da Graca Derengowski Fonseca & Mota, 2018; Dokur & Uysal, 2018; Huang et al., 2018; McDowell et al., 2017; Prats et al., 2018; Tur-Porcar et al., 2018; Zhang et al., 2018). Bibliometric analysis is frequently used to evaluate the quality of scholarly work because subjective factors do not influence it. The citation has been used as an index for assessing the value of literature. To a certain extent, the high citation articles are of more significant impact and analysis of top-cited original articles may show useful information on the specific research field (Fardi et al., 2011). We also ranked the top 10 most-cited articles about immunotherapy of sepsis and summarized their basic information such as title, publication year, journals, first author, country, and Scopus citations (Table 1). The significance of this bibliometric analysis is that it is the first time to use the method to obtain global research information in the field of immunotherapy for sepsis. We were astonished to find that 70% of the 10 articles were published in top international journals, including JAMA (The Journal of the American Medical Association), New England Journal of Medicine, Nature Medicine, and Nature Reviews Immunology. In 2013, the two critical care journals that published the same sepsis guidelines together and the number of citations for these guidelines ranked first in the field. Moreover, the number of their median citations is 1,175, which indicated that they expounded the critical scientific research achievements, so they have attracted wide attention.

After this study, we confirmed the dominant influence of the USA in the field of immunotherapy for sepsis. The number of publications from the USA was far more than any other country, accounting for about 41%, and was more than the number of articles from the second country to the sixth (China, Germany, UK, France, and the Netherlands) (Table 2). Also, as previously analyzed, the US published eight of the top 10 most cited articles. It was revealing that the USA produced both large quantities and high impact articles.

Additionally, Pearson correlations pointed out that countries with high GDP tend to make more contributions to the field of immunotherapy for sepsis. The reason for this pattern is usually that countries with high GDP have relatively abundant research funds, better national policies, sound medical infrastructure (e.g., advanced intensive care facilities) and excellent medical staff reserved (Reinhart et al., 2017). In this case, if the long-term steady growth of publications in this area is to be maintained, governments need to substantially increase investment and work to improve health infrastructure, especially in low- and middle-income countries (Saxena et al., 2006). Only with the support of these robust research findings can our policymakers and health care practitioners use this as a basis for intervention and improvement in clinical practice (Peters Van Ton et al., 2018; Van der Poll, 2001).

In general, this bibliometric analysis provides a macroscopic perspective for the immunotherapy of sepsis, which can be used to predict future research trends, guide funding policies, and provide practical and useful help for the decision-making of government workers and medical researchers who are interested in this field.

Of course, there are some limitations to this study, and we need to be aware of them. First, we only searched the Scopus database so the single data search strategy may have resulted in incomplete data collection. Second, since there is always some controversial literature, some researchers do not believe that highly cited research is equal to high-quality research. We also did not account for self-citations in the analysis. Also, detailed GDP investment in this area may be representative, but it is a complex task and challenging to complete. However, we still believe that such a large amount of macro data and such detailed literature analysis are enough to represent the development trend of this research field. Finally, we believe that researchers can ignore a tiny number of potentially missing publications and that our results are sufficient to represent the trends in this field.

## Conclusions

To sum up, this is the first bibliometric study evaluating the worldwide productivity in the field of immunotherapy for sepsis and we found that countries with high GDP tend to make more contributions to the field. The number of articles published in this field has been growing, especially in the last decade, when the number of articles has exploded. Whether measured by the total number of publications or the impact of the articles, the US is the strongest. Moreover, the journal that published the highest number of articles was Critical Care Medicine. The results of this series of analyses are intended to help policymakers and relevant researchers promote further development in this field.

## Supplemental Information

10.7717/peerj.7116/supp-1 Supplemental Information 1 Volume of publications per year.

10.7717/peerj.7116/supp-2 Supplemental Information 2 Statistics by country and region’s GDP and scientific research output.

10.7717/peerj.7116/supp-3 Supplemental Information 3 The Spearman rank correlation test.

## Additional Information and Declarations

### Data Availability

The following information was supplied regarding data availability: Raw data is available as Supplemental Files.
