# Supplementary figures and images for: Worldwide scientific productions with immunotherapy of sepsis: a bibliometric analysis
Source: PeerJ. 2019 Jun 17;7:e7116. doi: 10.7717/peerj.7116 (PMC6585897; doi:10.7717/peerj.7116)

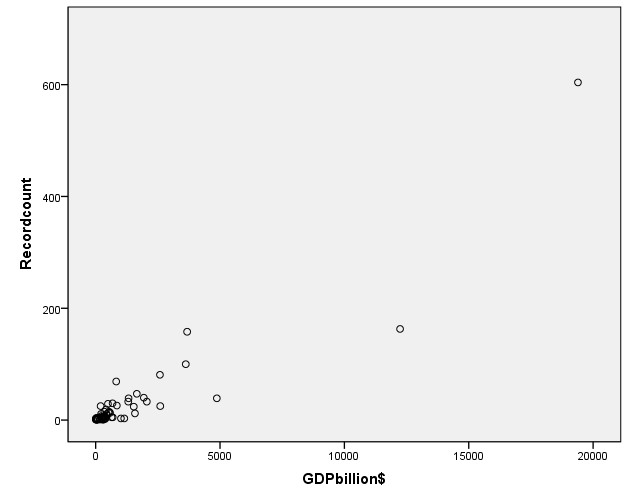

Supplement: Supplemental Information 3 [file peerj-07-7116-s003.png]
